# Association and causality between diabetes and activin A: a two-sample Mendelian randomization study

**DOI:** 10.3389/fendo.2024.1414585

**Published:** 2024-08-29

**Authors:** Mengqiao Wang

**Affiliations:** Department of Epidemiology and Biostatistics, School of Public Health, Chengdu Medical College, Chengdu, Sichuan, China

**Keywords:** Mendelian randomization, diabetes, activin A, genome-wide association studies, causal inference

## Abstract

Activin A, a cytokine belonging to the transforming growth factor-beta (TGF-β) superfamily, mediates a multifunctional signaling pathway that is essential for embryonic development, cell differentiation, metabolic regulation, and physiological equilibrium. Biomedical research using diabetes-based model organisms and cellular cultures reports evidence of different activin A levels between diabetic and control groups. Activin A is highly conserved across species and universally expressed among disparate tissues. A systematic review of published literatures on human populations reveals association of plasma activin A levels with diabetic patients in some (7) but not in others (5) of the studies. With summarized data from publicly available genome-wide association studies (GWASs), a two-sample Mendelian randomization (TSMR) analysis is conducted on the causality between the exposure and the outcome. Wald ratio estimates from single instruments are predominantly non-significant. In contrast to positive controls between diabetes and plasma cholesterol levels, inverse-variance-weighted (IVW), Egger, weighted median, and weighted mode MR methods all lead to no observed causal link between diabetes (type 1 and type 2) and plasma activin A levels. Unavailability of strong instruments prevents the reversal MR analysis of activin A on diabetes. In summary, further research is needed to confirm or deny the potential association between diabetes and plasma activin A, and to elucidate the temporal incidence of these traits in human populations. At this stage, no causality has been found between diabetes and plasma activin A based on TSMR analysis.

## Introduction

Activins/inhibins, among the 33 members of the transforming growth factor-beta (TGF-β) superfamily, have emerged as intriguing regulators of diverse physiological functions (1). Initially discovered based on their ability to stimulate (activins) or inhibit (inhibins) follicle-stimulating hormone (FSH) secretion in the pituitary gland (2), this branch of TGF-β superfamily assembles by the dimerization of subunits, with activins being homodimers or heterodimers of various β subunits (βA, βB, βC, and βE, respectively, encoded by *INHBA*, *INHBB*, *INHBC*, and *INHBE* genes), and inhibins being dimers of one β subunit and one α subunit (encoded by *INHA* gene) (3). Activin A is the most prominent member of the activins/inhibins and exerts its effects as a cytokine by binding to specific surface receptors and activating downstream signaling pathways, ultimately modifying expression profiles and eliciting cellular responses. Mechanistically, activin A binds to and activates a specific heterotetrameric complex of two type I (ActRI) and two type II (ActRII) transmembrane serine/threonine kinase receptors (3), signaling through multiple parallel downstream pathways including 1) the SMAD2/3/4 complex, 2) the ERK and JNK kinases, and 3) the Wnt pathway, all ultimately leading to the regulatory transcription of target genes. In addition, other TGF-β members (e.g., Nodal, BMP, GDF) could compete with activin A for binding to the specific receptors, highlighting the interconnected nature as well as the complex cross talk (3). The pleiotropic flexibility and versatility in activin A signaling involves the pathway in essential cellular processes, including but not limited to embryonic development, stem cell differentiation, cell proliferation, and inflammation. Consequently, dysregulation of activin A signaling has been implicated in the pathogenesis of various diseases (1), e.g., the progression of cancers, pulmonary/liver fibrosis, and development disorders.

Diabetes is a worldwide serious chronic disease characterized by unhealthy high blood glucose levels due to either insulin deficiency (type 1 diabetes, T1D) or insulin resistance (type 2 diabetes, T2D). Interestingly, there has been growing evidence from both model organisms and human studies on a potential link between activin A signaling and the mechanism of diabetes. Treatment with activin A effectively promotes regeneration of β cells and improves glucose metabolism in newborn rats treated with streptozotocin (an experimental model for induction of diabetes in rats) (4). In transgenic mice, activins and their receptors are upregulated in duct epithelial cells during islet differentiation, and blockade of activin A signaling inhibits the differentiation of pancreatic β cells (5). Being the most highly expressed of the TGF-β superfamily in human β-cells, activin A acts as a secretory protein with paracrine and autocrine effects on human islet cells (6), improving their viability in the cultured condition and stimulating insulin secretion in islets from T2D donors (7). Activin A levels are dysregulated in relation to obesity, and it was argued that potentially, the excess adipose tissue in obese individuals releases inflammatory activin A leading to insulin resistance and impaired glucose regulation (8). In diabetic patients, activin A levels are increased in the blood and pancreatic tissue of affected individuals (9, 10), possibly a result of the ongoing inflammatory responses triggered by the immune system’s attack on the innate β-cells. The implication of multifunctional cytokine activin A in the development and progression of diabetes highlights its potential role in endocrinological metabolism.

The link between diabetes and insulin is undeniable and intrinsically causal. However, the potential association between diabetes and activin A is less elucidated. Even if such a link exists, whether the association between diabetes and activin A is causal remains unknown. This has important implications since etiology aims at the discovery of not just risk factors but causal links. A causal relationship, if true, would directly imply alleviating diabetic development with molecular cytokine inhibitors and cellular receptor antagonists, thus making the activin A pathway a precise therapeutic target. Causality test between a pair of exposure and outcome is best inferred from randomized controlled trials (RCTs), balancing potential confounders between exposure groups by way of individual random assignment. Observational studies are better suited for association discoveries than causal inferences due to unadjusted confounding effects. However, adapting the usage of single-nucleotide polymorphisms (SNPs) from observational genome-wide association studies (GWASs) as instrumental variables (IVs), Mendelian randomization (MR) is a technique used in genetic epidemiology to investigate causal relationships between modifiable exposures and phenotypic outcomes (11), taking advantage of genetic variants that are randomly assigned at conception to mimic an observational quasi-RCT. A two-sample MR (TSMR) study extracts IVs associated with the exposure and with the outcome from two non-overlapping respective studies and serves as the core methodology in this study to investigate the presence or absence of causality between the exposure of diabetes and the outcome of plasma activin A levels. The results would provide supporting evidence in shaping our understanding of how the activin A signaling pathway may relate to human health in the perspective of diabetes.

## Methods

### Ethics

This study does not involve laboratory experiments or social surveys. The study protocol has been reviewed and approved by the Chengdu Medical College Research Ethics Committee (REC_2022–12).

### Data source

Activin A gene/protein sequences from various species are retrieved from the NCBI database (https://www.ncbi.nlm.nih.gov/), and protein structure files are downloaded from the PDB database (https://www.rcsb.org/). Tissue-specific *INHBA* RNA-seq data are accessed from the GTEx database (12) (https://www.gtexportal.org/). Summarized GWAS data of the exposures and the outcomes are retrieved from the IEU Open GWAS Project (13) (https://gwas.mrcieu.ac.uk/).

### Sequence alignment and analysis

Activin A protein sequences from eight species (human [*Homo sapiens*], house mouse [*Mus musculus*], dog [*Canis lupus familiaris*], domestic cat [*Felis catus*], zebrafish [*Danio rerio*], giant panda [*Ailuropoda melanoleuca*], goldfish [*Carassius auratus*], and common frog [*Rana temporaria*]) are downloaded and compared. Multiple-sequence alignment of protein sequences is conducted using the MUSCLE algorithm (14). Pairwise distances between two species are calculated using the identity matrix. The phylogenetic tree is constructed using the neighbor joining algorithm.

### Systematic literature review

An exhaustive literature search is conducted using key words of *activin A* and *diabetes* in the PUBMED database using the yearly time range (1995–2023) and limiting selection of research articles on studies of human populations. A total of 12 between-group comparisons from 10 papers are finally included in the systematic review, with group design, sample size, plasma activin A levels, and hypothesis-test *P* value retrieved and recorded.

### Mendelian randomization

Summarized data of SNPs associated with the corresponding traits from published or released GWASs are downloaded from the IEU Open GWAS database (13). GWASs of large sample sizes, high quality, and recent publication dates are selected for the following traits: T1D (15) (ebi-a-GCST90014023, 9 cohorts combined, European population, *n*_case_ = 18,942, *n* = 520,580), T2D (16) [ebi-a-GCST90018926, 3 cohorts combined, East Asian (28.5%) and European (71.5%) combined populations, *n*_case_ = 38,841, *n* = 490,089], LDL (17) [ieu-b-110, UK Biobank, European population, *n* = 440,546), HDL (17) (ieu-b-109, UK Biobank, European population, *n* = 403,943]; for plasma activin A levels, there are only two GWASs [#1. “eqtl-a-ENSG00000122641, European population, *n* = 30,765” and #2. “prot-c-2748_3_2, European population, *n* = 3,080” (18)] currently available, and both GWASs are included in this study for parallel analysis. A threshold of *P* value under 5 × 10^−8^ for the exposure and a clumping threshold of 0.001 for linkage disequilibrium in a 10,000-kb clumping distance window are used to select IVs for the exposure. IV strength is evaluated by the F-statistic (>10 used as a general indication for strong instruments), and IVs missing in the outcome are replaced by proxy SNPs when possible. All associations have been harmonized. Causality between exposure and outcome based on a single IV is estimated by the Wald ratio method. For weighted effects from multiple instruments, four MR methods of inverse variance weighted (IVW), Egger, weighted median, and weighted mode are applied. Post-MR analysis includes the heterogeneity test (Q statistic) and the pleiotropy tests [MR-Egger intercept, and MR-PRESSO (19)].

### Data analysis

All data analysis is conducted in the R environment (version 4.3.1). MR analysis is primarily conducted with the *TwoSampleMR* (20) package. Data visualization is prepared using the *ggplot2* and *r3dmol* (21) packages and extensions. A significance level of α = 0.05, when not otherwise stated, is used with regard to the *P* value or the false discovery rate (FDR). The Benjamini-Hochberg procedure is used to control the FDR.

## Results

### Activin A as a cytokine

**Figure 1:**
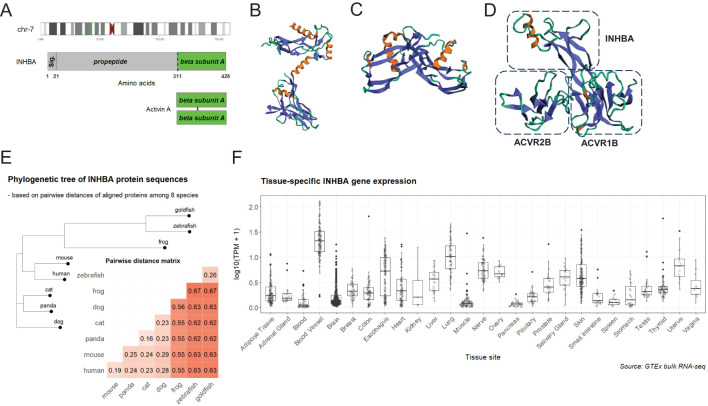
Summary of activin A as a highly-conserved polypeptide. **(A)** (upper) *INHBA* gene locates to the chromosome 7p14.1 region (green bar in the ideogram); (middle) full-length proprotein encoded by *INHBA* contains a conserved cleavage site (blue dashed line) targeted by furin-like proteases; (lower) activin A is a homodimer of two βA subunits linked by a disulfide bond. **(B)** Structure of the monomer proprotein (22 )(5HLY). **(C)** Structure of homodimer of activin A (23 )(2ARV). **(D)** Structure of a complex of activin A binding to ActRII-ActRI receptors (24 )(7OLY); only a one-sided part of the complex is displayed. **(E)** Aligned protein sequences among eight species (human, mouse, panda, cat, dog, zebrafish, goldfish, and frog) are compared for pairwise distances using identity matrix and visualized as an annotated heatmap. A phylogenetic tree is constructed with the neighbor joining algorithm based on pairwise distances among eight species. **(F)** Bulk RNA-seq transcriptional levels (transcripts per kilobase million, TPM) of *INHBA* among GTEx samples of different tissues are compared in boxplots. Tissue sites are ordered alphabetically.

The βA subunit (NP_002183.1) encoded by the *INHBA* gene at the 7p14.1 region is initially produced as a precursor proprotein (426 amino acids in human) composed of a signal peptide, an amino-terminal prodomain, and a carboxy-terminal mature domain (**Figure 1A**). Upon cleavage by furin-like proteases at a consensus RX(K/R)R motif, the mature domains dimerize in the endoplasmic reticulum to form a homodimer (activin A) linked by a single disulfide bond and finally navigate for secretion and release as a cytokine (3). Biophysical 3D structures have been solved for the proprotein (22) (5HLY, **Figure 1B**), the homodimer of activin A (23) (2ARV, **Figure 1C**), and a complex of activin A binding to ActRII-ActRI receptors (24) (7OLY, **Figure 1D**), illustrating the cytokine and receptor conformational landscape of the signaling pathway. In agreement with its essential roles in development and physiology, activin A is a highly conserved protein by sequence alignment among eight eukaryotic species (**Supplementary Figure S1**), and the phylogenetic tree based on pairwise distances reasonably matches the evolutionary history (**Figure 1E**). Being a cytokine, activin A is universally expressed in different parts of the human body, particularly highly expressed in tissues including adipose tissue, blood vessel, brain, lung, and nerve (**Figure 1F**). The expression pattern of activin A *in vivo* is reflective of previous findings of its involvement in cellular processes such as embryonic development, stem cell differentiation, and cell metabolism.

### Evidence-based review of association between the status of diabetes and activin A levels

While the association between diabetes and insulin has been well established as a fact and universally accepted as causal, there has been long-standing interest in characterizing the potential association between diabetes and various cytokines (including activin A). Retrieving and annotating published literatures, an evidence-based review is conducted to summarize the discoveries of plasma activin A levels in human studies conditioned on the diabetic status. Technically, activin A in plasma is assayed by antibody-based enzyme-linked immunosorbent assay (ELISA), most likely in the hundreds of pg/ml unit. A lack of conclusive agreement is revealed in the contradictory results of between-group comparisons in the summarized studies (**Table 1**): in five studies, activin A is not associated with diabetic status (thus unable to discriminate diabetic risk in individuals), but in the other seven studies, hypersecretion of activin A is present in diabetic patients compared with the healthy controls. Such disagreement in human populations is reminiscent of a previous review finding that “*studies on effects of activins on glucose homeostasis have produced disparate results in vivo and in vitro*” (1). The relatively low abundance and large fluctuation of activin A as a cytokine, together with the heterogeneity in diagnostic assays, disease severity, and diabetic subtypes, may all contribute to the unresolved inconsistence discovered in this systematic review. However, there may appear a supporting trend of statistically significant association of diabetes and circulating activin A in larger sample-size studies (**Table 1**). On the other aspect, confounders such as age and BMI are generally unconsidered or unadjusted, so analysis in these studies just reports the hypothesis test results of raw activin A levels between diabetic and control groups. Therefore, whether diabetes is truly associated with plasma activin A in human populations remains a puzzle, and its resolution requires scientifically more targeted observational/experimental evidence.

**Table 1:** Evidence-based summary of published literatures (ordered decreasingly by publication year) on their findings of plasma activin A levels compared between diabetic cases and healthy controls.

Study	Comparison group (sample size)	[Act A]_control_ (pg/ml)	[Act A]_case_ (pg/ml)	*P*	Note
Chauhan 2022 (25) Sylow 2020 (26) *Bian 2019 (* 9) *Bian 2019 (* 9) Kuo 2018 (10) Kuo 2018 (10) Naf 2014 (27) Chen 2013 (28) Ueland 2012 (29) Wu 2012 (30) Weigert 2009 (31) Petraglia 1995 (32)	Prediabetes (60) *vs.* control (60) T2D (10) *vs.* control (12) Diabetes (46) *vs.* control (12) Diabetes (160) *vs.* control (64) Prediabetes (168) *vs.* control (215) Diabetes (87) *vs.* control (215) Diabetes^#^ (78) *vs.* control (129) T2D (78) *vs.* control (14) T2D (102) *vs.* control (20) T2D (36) *vs.* control (39) T2D (30) *vs.* control (27) Diabetes^#^ (9) *vs.* control (7)	159.9(28.0)^!^ ~200^ 355.4 ± 79.8 241.3 ± 67.6 491.2 ± 165.3 491.2 ± 165.3 1.75(1.01)^!^ 315(~150^) 0.47(0.17)^!^ 90.1 ± 24.8 254.2 ± 187.5 25.4 ± 27.8^!^	263.6(52.4)^!^ ~200^ 508.1 ± 190.4 392.2 ± 169.4 559.0 ± 178.5 572.7 ± 167.0 1.78(1.24)^!^ 293(~100^) 0.56(0.32)^!^ 98.4 ± 31.6 231.9 ± 123.9 52.4 ± 23.3^!^	<0.001* >0.05 0.007* <0.0001* 0.007* <0.0001* 0.50 0.42 <0.05* >0.05 <0.05*	^!^ng/ml ^Unreported Mayo cohort Galway cohort ^#^Gestational;^!^ng/ml ^Unreported ^!^ng/ml ^#^Gestational;^!^μg/l

[Act A]: plasma activin A levels, shown as mean ± SD or median(IQR).

^: values not reported explicitly but inferred from figures.

*: *P* < 0.05.

### A two-sample MR study of diabetes and plasma activin A

**Figure 2:**
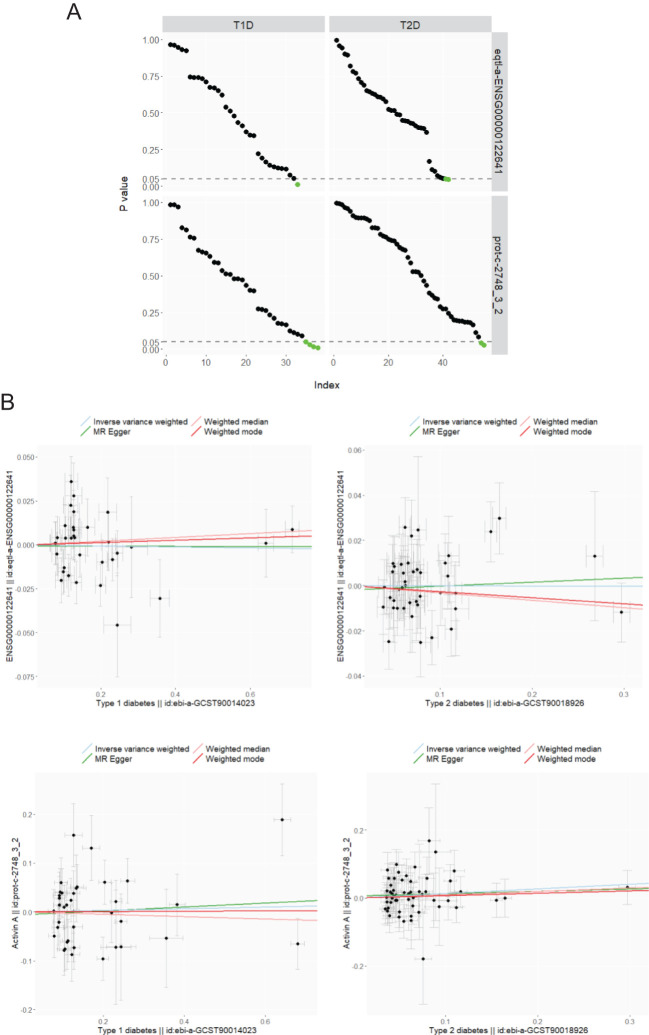
MR analysis of diabetes (T1D: ebi-a-GCST90014023; T2D: ebi-a-GCST90018926) on plasma activin A levels (#1. eqtl-a-ENSG00000122641 and #2. prot-c-2748_3_2). **(A)** *P* values from the Wald ratio estimates of single IVs between the exposure (columns) and the outcome (rows) are ordered decreasingly and visualized upon the IV index (number of IVs as 33, 42, 38, and 55 varies for each panel), and those under 0.05 are displayed in green color. **(B)** MR of T1D (left column) and T2D (right column) on plasma activin A levels of eqtl-a-ENSG00000122641 (upper row) and prot-c-2748_3_2 (lower row). Scatter plots: association of IVs with the exposure and the outcome (βs for point coordinates, and standard errors for error bars); line plots: multiple-IV MR estimates of IVW, Egger, weighted median, and weighted mode.

MR analysis is applied to investigate whether a causal link exists between the exposure of diabetes and the outcome of plasma activin A levels, for which associations of SNPs are retrieved from separate non-overlapping GWASs (see *Methods* for details). Given the power conferred by large sample sizes, a decent number of strong IVs are selected for both T1D (minimal F-statistic of 30.7) and T2D (minimal F-statistic of 29.6). An extensive search reveals the availability of only two GWASs for the phenotype of plasma activin A levels (#1 and #2), and both are included in the TSMR for parallel analysis. Therefore, a total of four exposure-outcome pairs are included in this study (T1D-activin A #1, T2D-activin A #1, T1D-activin A #2, and T2D-activin A #2). In total, nine SNPs with significant Wald ratio estimates (**Supplementary Figure S2**, left panel: 1 SNP, right panel: 2 SNPs; **Supplementary Figure S3**, left panel: 4 SNPs; right panel: 2 SNPs) have *P* values in the range of 0.01–0.05, revealing mild single-IV effects. In contrast, most qualified IVs display non-significant single-instrument causal estimates between T1D and plasma activin A, and between T2D and plasma activin A (**Supplementary Figures S2**, **S3**, **Figure 2A**), indicating a potential non-presence of causal link. For meta-analysis of causal estimates from single instruments, all four MR methods (IVW, Egger, weighted median, and weighted mode) reveal a combined non-significance of causal inference between diabetes (either T1D or T2D) and plasma activin A levels (**Figure 2B**, **Table 2**). As positive controls, such MR methods reveal causality on previously observed associations between diabetes and plasma cholesterols (33) (especially LDL cholesterol) (**Supplementary Table S1**). For post-MR analysis in all four “diabetes-activin A” pairs, heterogeneity test of the IVW method reveals Cochran’s Q statistic close to the corresponding degree of freedom and fails to detect significant heterogeneity among multiple instruments with *P* values in the range of 0.08–0.91 (all >0.05); pleiotropy test of whether the MR-Egger intercept is different from zero reports *P* values in the range of 0.62–0.89 (all >0.05), and pleiotropy test by the MR-PRESSO global test reports *P* values in the range of 0.07–0.98 (all >0.05), thus suggesting that there exists no detectable horizontal pleiotropy in the causal analysis (a key MR assumption). Therefore, this study conducts a robust TSMR analysis in which no causal link is observed between the exposure of diabetes and the outcome of plasma activin A levels in human.

**Table 2:** Two-sample MR analysis of T1D and T2D (exposures) on plasma activin A levels (outcome, two GWASs used).

	Method	*n*	*β* (se)	*P*		Method	*n*	*β* (se)	*P*
**T1D on activin A #1**	IVW Egger Weighted median Weighted mode	33 33 33 33	−0.0028 (0.0122) −0.0006 (0.0200) 0.0108 (0.0149) 0.0066 (0.0154)	0.82 0.98 0.47 0.67	**T1D on activin A #2**	IVW Egger Weighted median Weighted mode	38 38 38 38	0.0169 (0.0483) 0.0400 (0.0815) −0.0243 (0.0657) 0.0036 (0.0718)	0.73 0.63 0.71 0.96
**T2D on activin A #1**	IVW Egger Weighted median Weighted mode	42 42 42 42	−0.0012 (0.0226) 0.0176 (0.0444) −0.0328 (0.0376) −0.0268 (0.0423)	0.96 0.69 0.38 0.53	**T2D on activin A #2**	IVW Egger Weighted median Weighted mode	55 55 55 55	0.1362 (0.0889) 0.0685 (0.1610) 0.0988 (0.1410) 0.0716 (0.1252)	0.13 0.67 0.48 0.57

Exposure: T1D (ebi-a-GCST90014023), T2D (ebi-a-GCST90018926).

Outcome: plasma activin A (#1. eqtl-a-ENSG00000122641 in the left panel, and #2. prot-c-2748_3_2 in the right panel).

### Reverse MR study of plasma activin A and diabetes

The potential association between diabetes and plasma activin A levels could be in the opposite direction, in which dysregulation of activin A secretion leads to higher risk of diabetes. This is possible given that in two independent studies from the literature review (10, 25), even prediabetic individuals display significantly higher activin A levels compared with the controls (**Table 1**). Therefore, it is tempting to conduct a reverse MR using plasma activin A as the exposure and the diabetic status as the outcome. However, such analysis becomes impractical as there are no strong IVs (eQTLs) of activin A in the only two available GWASs: the lowest *P* value and FDR of SNPs associated with plasma activin A levels are respectively 0.0001 and 0.79 in ebi-a-GCST90018926, and 2.8 × 10^−6^ and 0.96 in prot-c-2748_3_2 (**Supplementary Figure S4**). The absence of SNPs with *P* value <5 × 10^−8^ or FDR <0.05 in both GWASs renders the unavailability of IVs that are strongly associated with the exposure (an essential MR assumption). Nevertheless, an exploratory reverse MR trial using top SNPs from these two GWASs as weak instruments leads to negative causal inference of plasma activin A as the exposure and diabetes as the outcome (results not shown given the absence of strong instruments).

## Discussion

The activin A signaling pathway is a key regulatory component of cellular development, growth, and metabolism. The first step in differentiation of pluripotent stem cells toward endoderm-derived cells/organs is differentiation into the definitive endoderm. In the milestone work that produces cultures enriched for definitive endoderm cells from differentiated human embryonic stem cell (hESC) lines, the presence of high-dose activin A in a low serum medium highlights the protein’s essential roles in the differentiation of embryonic progenitors of pancreatic cells. As evidence for its glucose-dependent insulinotropic action, human pancreatic islets cocultured with activin A and glucose exhibits a dose-dependent insulin secretion phenomenon, but such response is absent when cells are cultured with activin A without glucose (34). Diabetes is particularly suited to the approach of stem cell therapies for a variety of conditions, as transplantation of the stem-cell-based insulin-producing pancreatic β-cells or glucose-responsive islet-like organoids could potentially provide long-lasting therapy or even a cure (35, 36). Circulating activin A could be a secreted biomarker for diabetes. Its dysregulated expression in both T1D and T2D patients suggests that it could be used as an indicator of disease progression, response to therapy, and overall prognosis. Not only does activin A play a role in the context of diabetes progression, but also it is involved in the pathogenesis of diabetic complications including diabetic nephropathy, diabetic retinopathy, cardiovascular disease, and fibrosis *etc. *(3) Furthermore, chronic low-grade inflammation is observed in diabetes, and activin A, as a pro-inflammatory cytokine, has been recognized for its role in regulating inflammation and immune responses (37), which could perpetuate the destruction of β-cells in T1D and aggravates insulin resistance in T2D. Consequently, monitoring activin A levels in individuals with diabetes may help in identifying those at a higher risk of developing complications, allowing for early intervention and better outcomes.

Understanding the association between diabetes and activin A represents an intriguing area of research with significant clinical implications. Initially, a statistically significant difference of activin A levels is observed in diabetes-related model organisms and cellular cultures (4–7). In this study, the systematic review (not a meta-analysis due to the absence of certain study results and to the disparate outcome mean *vs.* median values in the original articles) suggests that the association between diabetes and activin A in human populations is somewhat ambiguous. This challenge may be due to several factors: 1) low abundance of activin A in plasma (generally less than 1 ng/ml); 2) lack of fast and accurate biochemical assays; 3) as a cytokine, activin A levels may vary in response to different physiological status; 4) the diabetic patients may be at different disease stages; 5) small sample sizes; and 6) only a single cross-sectional measurement but no temporal track of activin A levels. It is highly recommended in future to address the above issues in large cohort studies so that the question of whether diabetes and activin A are associated and if yes, which precedes the other, could both be answered conclusively.

For molecular epidemiology, the availability of only two medium-size GWASs on activin A renders the lack of strong eQTLs and thus the difficulty to accurately quantify its levels in plasma by use of genetic instruments. Activin A is not a commonly measured cytokine in blood assays, so large cohorts such as the UK Biobank, the FinnGen Project, and Biobank Japan do not have this phenotype on record and thus could not confer a solid GWAS. Large-sample GWAS on a certain trait is essential to map strongly associated SNPs and filter appropriate instruments to be used in MR analysis, e.g., on an MR study with plasma homocysteine as the exposure (38). Future large-scale studies on human populations are necessary to both genotype the genomes and measure activin A levels so that more accurate and robust GWASs would be available to conduct TSMR and conclude if any potential association between diabetes and activin A in the human population is causal or not. If yes, the temporal order (which precedes the other) needs to be carefully elucidated in bidirectional TSMR so that the potential causal directionality could be determined for the appropriate exposure-outcome model. In addition, more confounder-controlled or -matched observational evidence together with model-based biomedical experiments is essential to discover the precise mechanisms underlying the potential associative or even causal link.

Under no deviation from three model assumptions, TSMR using genetic IVs has been established as a key approach to estimate causality from non-random observational studies. This study explores diabetes as the exposure and plasma activin A as the outcome, and all four MR methods reveal non-present causality. These results have important implications in the interpretation of potential associations. For a non-causal association, activin A in blood samples could function as a biomarker for diabetes, allowing for early detection and monitoring of this complex chronic disorder, but activin A as well as its receptors could not be used as therapeutic targets. In contrast, for a causal relationship, diabetes may lead to up-regulation of activin A and thus elicits a cascade of cytokine-dependent signaling events to confer physiological and metabolic consequences, and in such cases, activin A and its receptors could be explored as interventional nodes to slow diabetic symptoms. Compared with the positive controls of causal links between diabetes and plasma cholesterol levels, the conclusion of absent causality between diabetes and activin A in this study is evident but may not be final primarily again due to the lack of trustful large-scale GWAS on the trait of plasma activin A levels.

The reversal causality is of equal interest, as dysregulated activin A levels may contribute to the incidence and progression of diabetes. However, such reverse MR analysis using plasma activin A as the exposure and diabetes as the outcome is impractical at this moment because of the abovementioned lack of strong instruments (eQTLs) for activin A. Again, when reliable large-scale GWASs in future map and identify appropriate SNPs as qualified instruments for activin A, bidirectional TSMR could be applied to answer the reversal causality question. If present, the potential causal link between activin A and diabetes would hold great promise for the development of new effective therapies, in which targeting activin A and its receptors could potentially restore normal β-cell function, improve insulin sensitivity, and reduce inflammation in diabetic patients. For example, REGN2477, a fully human monoclonal antibody that inhibits activin A but no other TGF-β superfamily members, has been preclinically tested in mice for the potential treatment of muscle atrophy (39). In contrast, if the causal link from activin A to diabetes is non-present, any preclinical or clinical trials aimed at treating diabetes by targeting the activin A pathway would be futile.

In conclusion, the presence or absence of reciprocal association and even causality between diabetes and activin A is a multifaceted and unresolved field. The association within human populations remains controversial from the perspective of systematic review of published literatures, and the TSMR causality estimates from diabetes to plasma activin A is indifferent from null. Further research is needed and recommended to elucidate the interplay between these two physiological traits.

## Data Availability

Publicly available datasets were analyzed in this study. This data can be found in the Methods section, which includes all the dataset references.

## Supplementary material

Supplementary Figure 1 Multiple sequence alignment (by the MUSCLE algorithm) of activin A (*INHBA*) protein sequences among eight species.

Supplementary Figure 2 MR estimates of diabetes (T1D: ebi-a-GCST90014023, left; T2D: ebi-a-GCST90018926, right) on plasma activin A levels (#1. eqtl-a-ENSG00000122641). In the forest plot: for single IVs, the Wald ratio estimates as well as the corresponding 95% confidence intervals are respectively displayed (black); for multiple IVs, the IVW, Egger, weighted median, and weighted mode MR estimates as well as their 95% confidence intervals are displayed (red).

Supplementary Figure 3 MR estimates of diabetes (T1D: ebi-a-GCST90014023, left; T2D: ebi-a-GCST90018926, right) on plasma activin A levels (#2. prot-c-2748_3_2). In the forest plot: for single IVs, the Wald ratio estimates as well as the corresponding 95% confidence intervals are respectively displayed (black); for multiple IVs, the IVW, Egger, weighted median, and weighted mode MR estimates as well as their 95% confidence intervals are displayed (red).

Supplementary Figure 4 Volcano plot of two GWASs of plasma activin A (above: ebi-a-GCST90018926, 18978 SNPs; below: prot-c-2748_3_2, 501403 SNPs). Y axis is in -log_10_(*P* value) (left panel) or -log_10_(FDR) (right panel); X axis is in *β* estimates of the association with activin A. For selection of strong IVs in MR, a *P* value <5x10^-8^ (on Y axis as 7.3) or FDR <0.05 (on Y axis as 1.3) is generally selected as the thresholds to filter SNPs in GWAS. None of SNPs meet the thresholds in these two GWASs.

Supplementary Table 1 Two-sample MR analysis of T1D and T2D (exposures) on LDL and HDL cholesterol levels (outcomes). Exposure: T1D (ebi-a-GCST90014023), T2D (ebi-a-GCST90018926); Outcome: LDL cholesterol (ieu-b-110) in the left panel, and HDL cholesterol (ieu-b-109) in the right panel. *: *P* < 0.05.
