# Supplementary material for: Association and causality between diabetes and activin A: a two-sample Mendelian randomization study
Source: Front Endocrinol (Lausanne). 2024 Aug 29;15:1414585. doi: 10.3389/fendo.2024.1414585 (PMC11393405; doi:10.3389/fendo.2024.1414585)
Supplement: Supplementary Figure 1 — Multiple sequence alignment (by the MUSCLE algorithm) of activin A (INHBA) protein sequences among eight species. [file DataSheet1.docx]

**Supplementary figures**

**Figure S1**


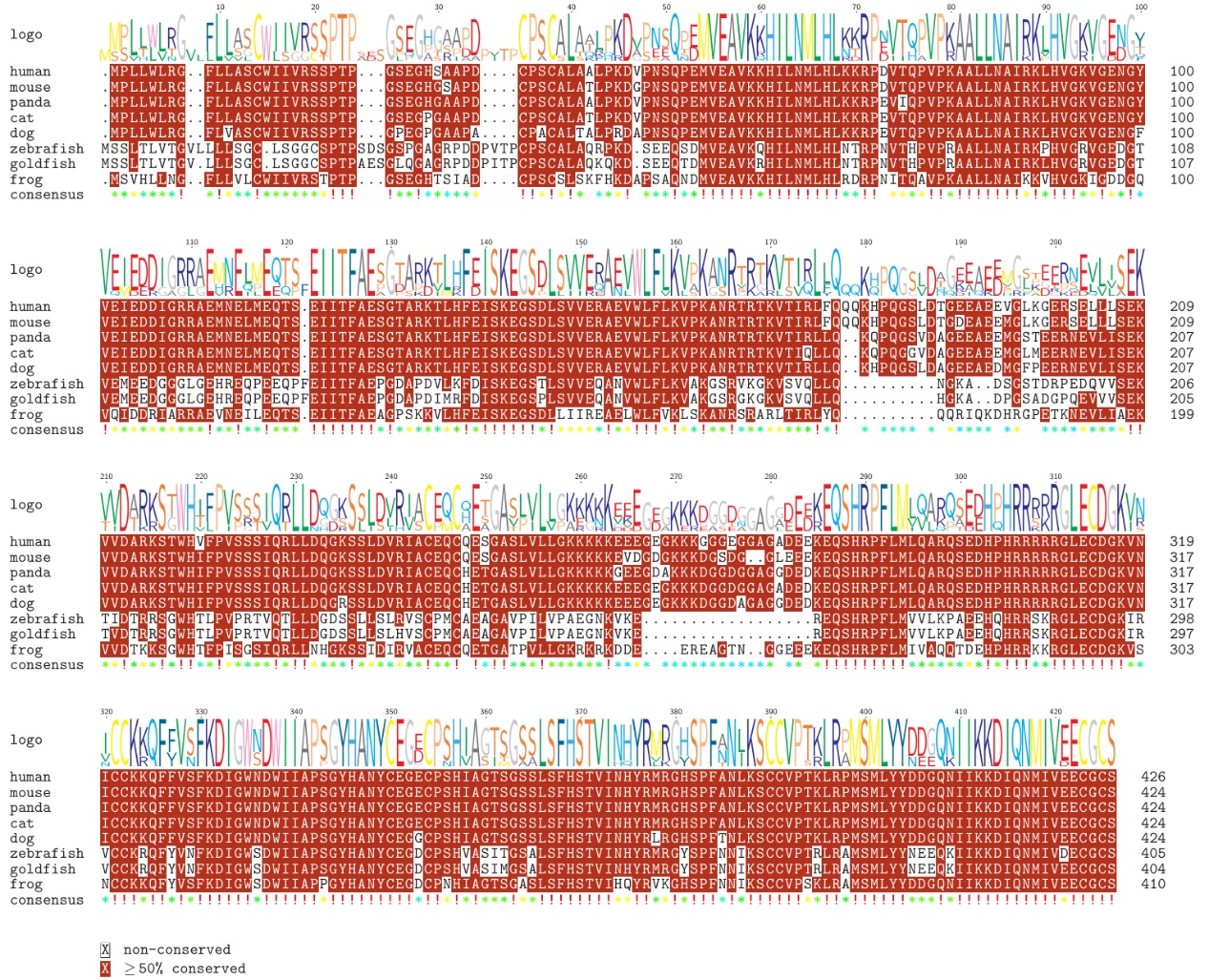


**Figure S1. Multiple sequence alignment (by the MUSCLE algorithm) of activin A (*INHBA*) protein sequences among eight species.**

**Figure S2**

**
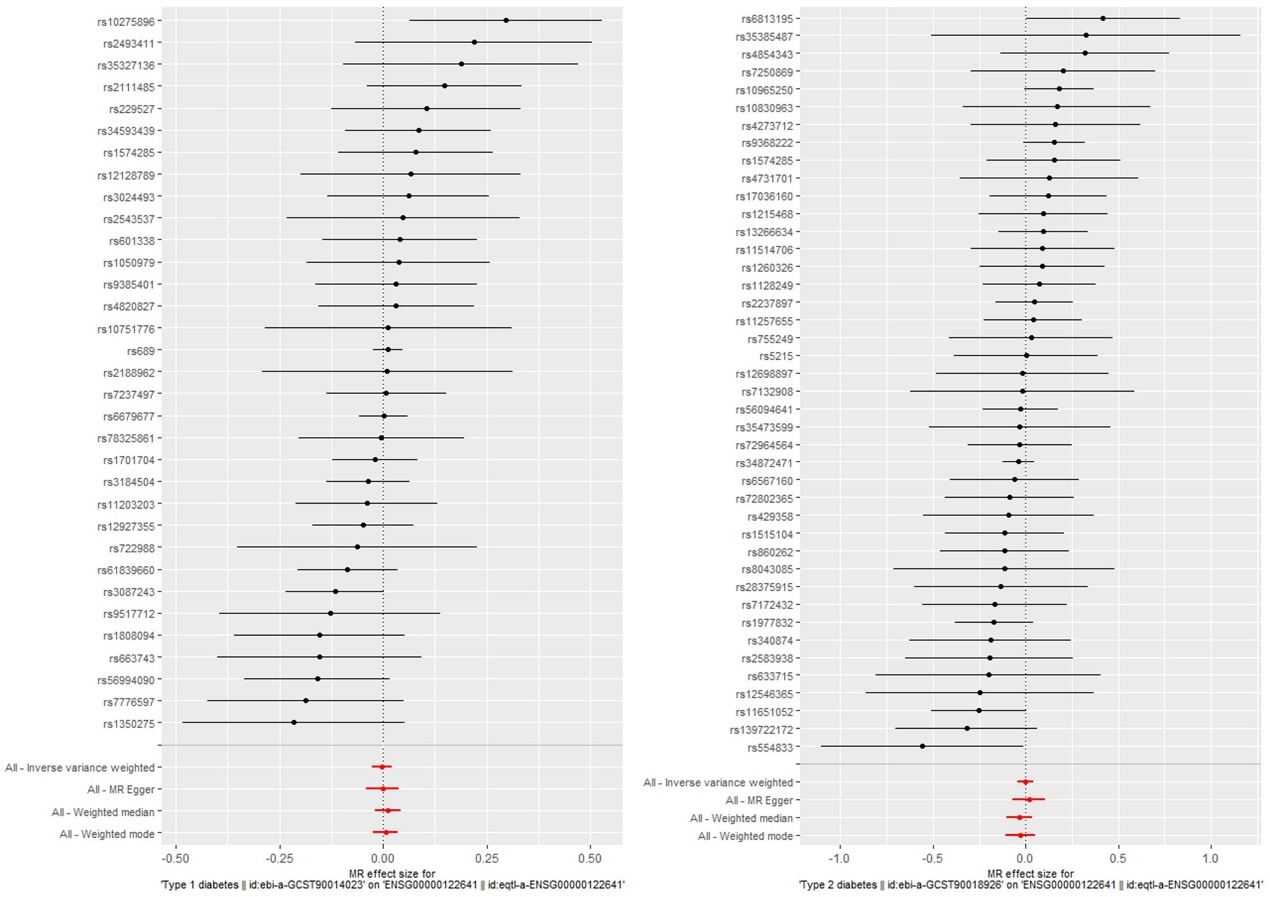
**

**Figure S2. MR estimates of diabetes (T1D: ebi-a-GCST90014023, left; T2D: ebi-a-GCST90018926, right) on plasma activin A levels (#1. eqtl-a-ENSG00000122641).** In the forest plot: for single IVs, the Wald ratio estimates as well as the corresponding 95% confidence intervals are respectively displayed (black); for multiple IVs, the IVW, Egger, weighted median, and weighted mode MR estimates as well as their 95% confidence intervals are displayed (red).

**Figure S3**

**
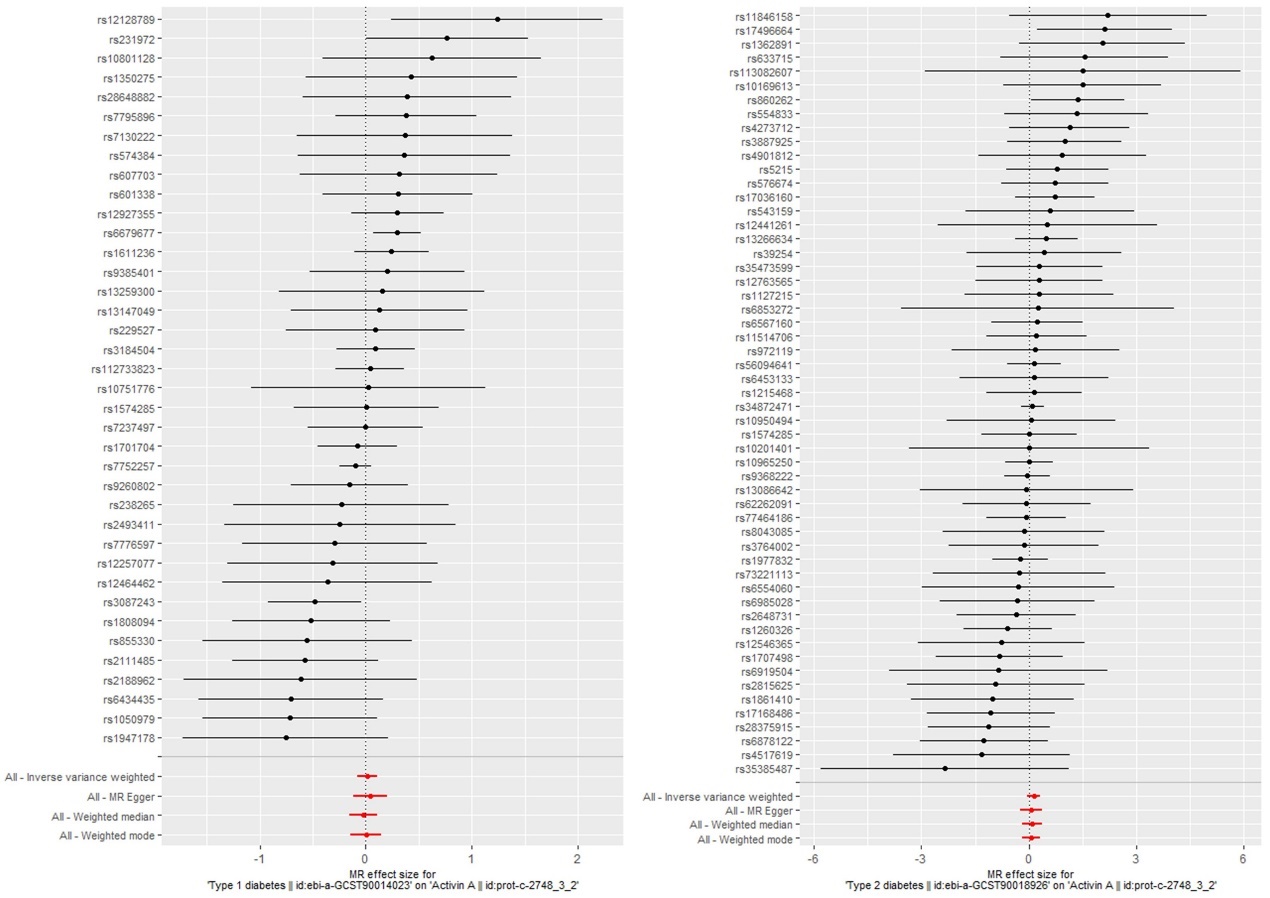
**

**Figure S3. MR estimates of diabetes (T1D: ebi-a-GCST90014023, left; T2D: ebi-a-GCST90018926, right) on plasma activin A levels (#2. prot-c-2748_3_2).** In the forest plot: for single IVs, the Wald ratio estimates as well as the corresponding 95% confidence intervals are respectively displayed (black); for multiple IVs, the IVW, Egger, weighted median, and weighted mode MR estimates as well as their 95% confidence intervals are displayed (red).

**Figure S4**


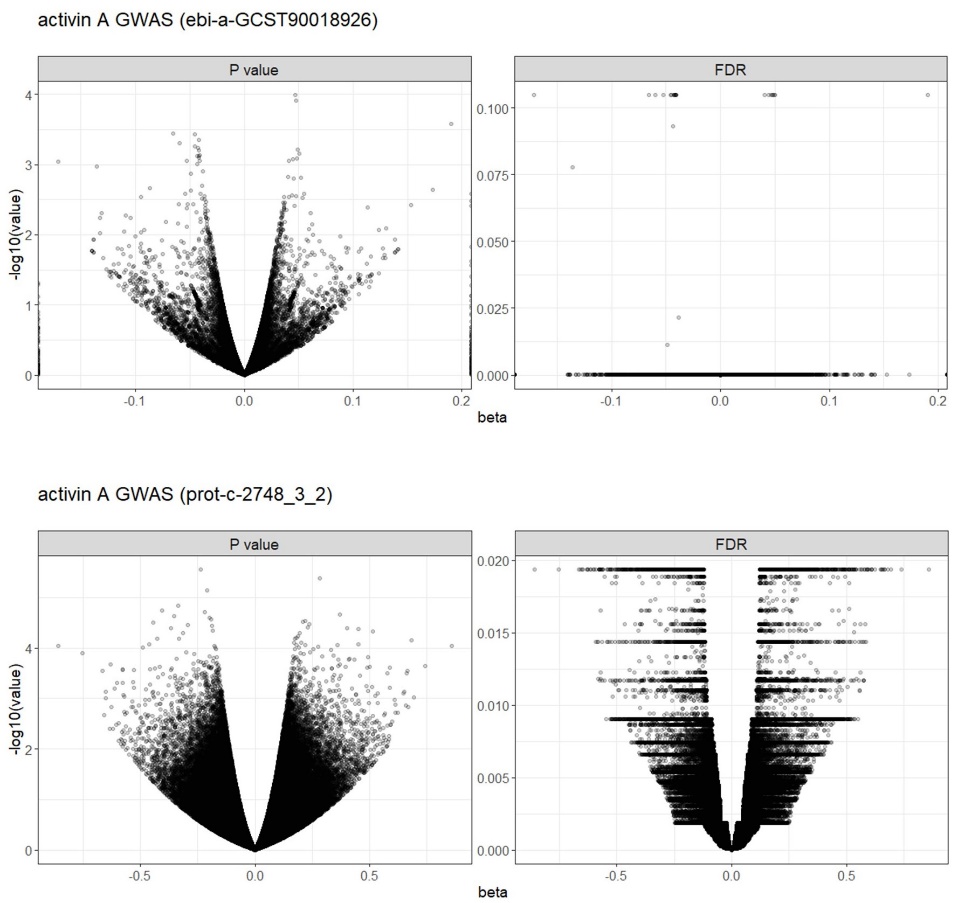


**Figure S4. Volcano plot of two GWASs of plasma activin A (above: ebi-a-GCST90018926, 18978 SNPs; below: prot-c-2748_3_2, 501403 SNPs).** Y axis is in -log_10_(*P* value) (left panel) or -log_10_(FDR) (right panel); X axis is in *β* estimates of the association with activin A. For selection of strong IVs in MR, a *P* value <5x10^-8^ (on Y axis as 7.3) or FDR <0.05 (on Y axis as 1.3) is generally selected as the thresholds to filter SNPs in GWAS. None of SNPs meet the thresholds in these two GWASs.
