# Supplementary material for: Association and causality between diabetes and activin A: a two-sample Mendelian randomization study
Source: Front Endocrinol (Lausanne). 2024 Aug 29;15:1414585. doi: 10.3389/fendo.2024.1414585 (PMC11393405; doi:10.3389/fendo.2024.1414585)
Supplement: Supplementary Table 1 — Two-sample MR analysis of T1D and T2D (exposures) on LDL and HDL cholesterol levels (outcomes). Exposure: T1D (ebi-a-GCST90014023), T2D (ebi-a-GCST90018926); Outcome: LDL cholesterol (ieu-b-110) in the left panel, and HDL cholesterol (ieu-b-109) in the right panel. *: P < 0.05. [file DataSheet2.docx]

**Supplementary tables**

**Table S1. Two-sample MR analysis of T1D and T2D (exposures) on LDL and HDL cholesterol levels (outcomes).**

|  | **Method** | ***n*** | ***β* (se)** | ***P*** |  | **Method** | ***n*** | ***β* (se)** | ***P*** |
| --- | --- | --- | --- | --- | --- | --- | --- | --- | --- |
| **T1D on LDL cholesterol** | IVW  Egger  weighted median | 84  84  84 | -0.010 (0.003)  -0.014 (0.004)  -0.014 (0.002) | 0.002*  0.002*  3*10^-15^* | **T1D on HDL cholesterol** | IVW  Egger  weighted median | 84  84  84 | -0.004 (0.003)  -0.0001 (0.004)  -0.005 (0.002) | 0.24  0.98  0.003* |
|  | weighted mode | 84 | -0.013 (0.002) | 6*10^-12^* |  | weighted mode | 84 | -0.002 (0.001) | 0.25 |
| **T2D on LDL cholesterol** | IVW | 170 | -0.051 (0.016) | 0.002* | **T2D on HDL cholesterol** | IVW | 170 | -0.054 (0.012) | 5*10^-6^* |
|  | Egger | 170 | -0.047 (0.034) | 0.17 |  | Egger | 170 | 0.016 (0.024) | 0.49 |
|  | weighted median | 170 | -0.034 (0.006) | 7*10^-9^* |  | weighted median | 170 | -0.016 (0.006) | 0.006* |
|  | weighted mode | 170 | -0.031 (0.007) | 7*10^-6^* |  | weighted mode | 170 | -0.005 (0.006) | 0.41 |

Exposure: T1D (ebi-a-GCST90014023), T2D (ebi-a-GCST90018926);

Outcome: LDL cholesterol (ieu-b-110) in the left panel, and HDL cholesterol (ieu-b-109) in the right panel.

*: *P* < 0.05.
